# *Phf8* loss confers resistance to depression-like and anxiety-like behaviors in mice

**DOI:** 10.1038/ncomms15142

**Published:** 2017-05-09

**Authors:** Ryan M. Walsh, Erica Y. Shen, Rosemary C. Bagot, Anthony Anselmo, Yan Jiang, Behnam Javidfar, Gregory J. Wojtkiewicz, Jennifer Cloutier, John W. Chen, Ruslan Sadreyev, Eric J. Nestler, Schahram Akbarian, Konrad Hochedlinger

**Affiliations:** 1 Department of Molecular Biology, Cancer Center and Center for Regenerative Medicine, Massachusetts General Hospital, Boston, Massachusetts 02114, USA; 2 Department of Stem Cell and Regenerative Biology and Harvard Stem Cell Institute, Cambridge, Massachusetts 02138, USA; 3 Howard Hughes Medical Institute, Chevy Chase, Maryland 20815, USA; 4 Friedman Brain Institute, Department of Neuroscience, Icahn School of Medicine at Mount Sinai, New York City, New York 10029, USA; 5 Center for Systems Biology, Massachusetts General Hospital, Harvard Medical School, Boston, Massachusetts 02114, USA; 6 Institute for Innovations in Imaging and Division of Neuroradiology, Department of Radiology, Massachusetts General Hospital, Harvard Medical School, Boston, Massachusetts 02114, USA

## Abstract

PHF8 is a histone demethylase with specificity for repressive modifications. While mutations of *PHF8* have been associated with cognitive defects and cleft lip/palate, its role in mammalian development and physiology remains unexplored. Here, we have generated a *Phf8* knockout allele in mice to examine the consequences of *Phf8* loss for development and behaviour. *Phf8* deficient mice neither display obvious developmental defects nor signs of cognitive impairment. However, we report a striking resiliency to stress-induced anxiety- and depression-like behaviour on loss of *Phf8*. We further observe misregulation of serotonin signalling within the prefrontal cortex of *Phf8* deficient mice and identify the serotonin receptors *Htr1a* and *Htr2a* as direct targets of PHF8. Our results clarify the functional role of *Phf8* in mammalian development and behaviour and establish a direct link between *Phf8* expression and serotonin signalling, identifying this histone demethylase as a potential target for the treatment of anxiety and depression.

## Abstract (web summary)

Mutation of the human gene *PHF8*, encoding a histone demethylase, is linked to cognitive defects but its role in development is unclear. Here, the authors show that *Phf8* deletion in mice causes no overt developmental defects but confers resilience to depression, likely through increased serotonin signalling.

Histone methylation at specific lysine residues plays a crucial role in transcriptional control by either facilitating or suppressing gene expression^1^. While methylation of the tail of histone H3 at lysine 4 (H3K4) and lysine 36 (H3K36) is generally associated with gene activation, methylation of H3 lysine 27 (H3K27), lysine 9 (H3K9) and histone H4 lysine 20 (H4K20) is associated with gene repression^2^,^3^. The methylation status of each of these residues is controlled by opposing enzymes, histone lysine methyltransferases and lysine demethylases. Notably, several lysine methyltransferases and demethylases have been directly implicated in embryonic development, cellular differentiation or cancer, underscoring the importance of histone methylation in health and disease^4^,^5^.

PHF8 is an X-linked histone demethylase containing a plant homeodomain and a catalytic Jumonji C (JMJC) domain. Interestingly, dysregulation of *PHF8* has recently been associated with developmental abnormalities and human disease. For instance, mutations in *PHF8* have been identified in a subset of patients with X-linked intellectual disability, which is often accompanied by cleft lip/palate^6^,^7^,^8^. Moreover, morpholino-mediated knockdown of *phf8* results in brain and craniofacial defects during zebrafish development^9^ whereas suppression of a *Phf8* homologue in *C. elegans* reportedly leads to behavioural abnormalities such as impaired locomotion^10^. Although, these knockdown studies in worms and zebrafish support an involvement of *Phf8* in embryonic development and adult behaviour, the functional role of this demethylase in a mammalian animal model following genetic perturbation of the endogenous *Phf8* locus remains unexplored.

PHF8 has been predominantly studied as a transcriptional activator through its ability to demethylate the repressive histone modifications H3K9me1/2, H3K27me2 and H4K20me1 (refs 9, 10, 11, 12). Consistently, ChIP-Seq data has revealed that PHF8 directly binds to ribosomal DNA and E2F1 targets in HeLa and U2OS cells, facilitating their activation, while knockdown of *Phf8* in these cell lines results in cellular growth defects^11^,^12^,^13^,^14^,^15^. It remains unclear, however, whether the observed proliferation defect in these transformed human cell lines is relevant for the reported developmental and behavioural abnormalities *in vivo*.

In addition to its role as a histone demethylase, PHF8 functions as a chromatin reader by directly binding H3K4me3 through a pocket in its plant homeodomain finger, enabling recruitment to chromatin and allosteric activation of demethylase activity^10^,^11^,^16^. More recent data indicate that PHF8 may also repress the transcription of certain target genes, although the underlying mechanisms remain unknown^17^.

Here, we revisit the molecular and functional role of *Phf8* in primary stem and differentiated cells, mammalian development and adult physiology by establishing a targeted deletion in mice. We find that *Phf8* null mice display neither developmental nor cognitive defects, but instead are resistant to anxiety- and depression-like behaviours. Moreover, we document that PHF8 directly binds to regulatory regions nearby the serotonin receptor genes *Htr1a* and *Htr2a* in the neocortex, providing a molecular basis for the observed phenotype.

## Results

### Targeted deletion of *Phf8* in mouse ESCs

**Figure 1:**
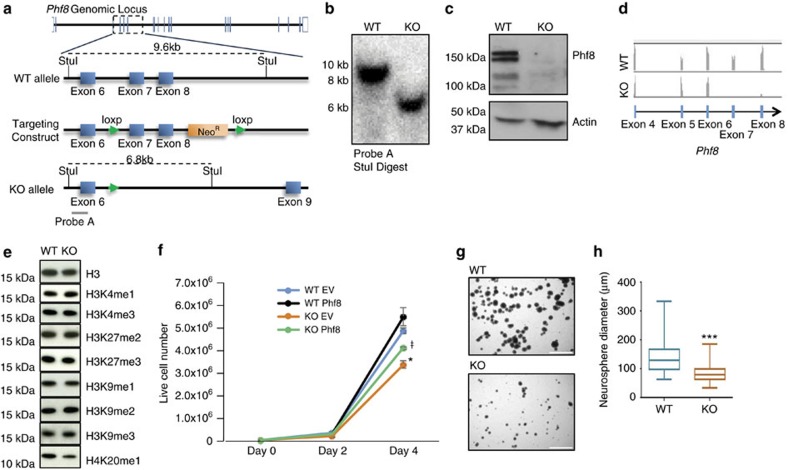
Generation and *in vitro* characterization of a *Phf8* null allele. (**a**) Diagram of the *Phf8*locus around exons 7 and 8 for wild type (WT) (top), targeted (middle) and knockout (KO) (bottom) alleles. (**b**) Southern blot analysis confirms deletion of exons 7 and 8 of *Phf8* in embryonic stem cells (ESCs) using StuI digest and probe A (see panel A). (**c**) Western blot analysis confirms loss of Phf8 protein in KO ESCs. (**d**) RNA-seq tracks from *Phf8* KO and WT ESCs show absence of reads for deleted exons 7 and 8 in KO sample. (**e**) Western blot analysis detecting indicated histone modifications in *Phf8* KO ESCs. (**f**) Growth curve for *Phf8* WT and KO ESCs transduced with either empty vector control (EV) or full length Phf8 cDNA (Phf8) lentiviral vectors. Error bars=s.e.m. *=*p*<0.05 for WT EV and KO EV. ^ŧ^=*p*<0.05 for KO Phf8 and KO EV (Student's *t*-test, two-tailed). (**g**) Representative images of *Phf8* WT and KO neurosphere cultures grown for 1 week from single cell suspensions. Scale bar: 750 μm (**h**) Quantification of neurosphere diameter 1 week after plating of single cell suspension. *=*p*<0.05, ^***^=*p*<0.001 (Student's *t*-test, two-tailed).

To generate a knockout (KO) allele for *Phf8*, we introduced *loxP* sites flanking exons 7 and 8 of the X-linked *Phf8* locus by conventional targeting in embryonic stem cells (ESCs). We chose these two exons as they encompass a critical portion of the catalytic JMJC domain, including a highly conserved histidine residue essential for the demethylation function of Phf8 (refs 11, 15) (Fig. 1a). Moreover, Cre-mediated deletion of these exons is expected to cause a frameshift mutation, disrupting the formation of full-length protein. Briefly, we electroporated the *Phf8* targeting construct into male V6.5 ESCs, exposed cells to drug selection, and identified correctly targeted clones by Southern blot analysis. Verified clones were subsequently electroporated with a vector transiently expressing CRE recombinase to generate *Phf8* KO ESCs, which were identified via Southern blot analysis (Fig. 1b).

We confirmed loss of *Phf8* in targeted ESCs by western blot analysis and RNA-sequencing (Fig. 1c,d). Notably, examination of H3K9me1/2/3, K3K27me2/3, H3K4me1/3 and K4K20me1 levels in *Phf8* KO and control ESCs using western blot analysis failed to show significant differences, indicating that loss of *Phf8* does not lead to a detectable global increase in these covalent modifications (Fig. 1e; Supplementary Fig. 1a), which is consistent with previous observations^9^.

### *In vitro g*rowth defects in *Phf8* ^*−/y*^ cells

Knockdown of *Phf8* reportedly causes growth defects in transformed cell lines^11^,^13^,^14^,^15^. To assess whether this phenotype is recapitulated in *Phf8* deficient primary cells at different stages of development, we determined proliferation rates of *Phf8* deficient murine embryonic fibroblasts (MEFs), neural progenitor cells (NPCs) and ESCs. Deletion of *Phf8* impaired the proliferative potential of MEFs, NPCs and ESCs compared to wild type (WT) controls (Fig. 1f–h; Supplementary Fig. 1b). Lentiviral overexpression of full-length Phf8 in *Phf8* KO ESCs partially rescued this phenotype (Fig. 1f; Supplementary Fig. 1c). These data indicate that the histone demethylase PHF8 regulates growth potential *in vitro* in multiple primary cell types, regardless of differentiation stage and tissue origin.

To identify possible downstream regulators of PHF8 that may cause the observed proliferation defect, we performed RNA-sequencing of *Phf8* deficient and WT NPCs and ESCs. Surprisingly, loss of *Phf8* did not lead to major expression differences in ESCs and NPCs, and unbiased hierarchical clustering was unable to separate KO and WT samples by genotype (Supplementary Fig. 2).

### *Phf8* ^*−/y*^ mice develop normally

Given the reported central nervous system and craniofacial defects in *phf8* knockdown zebrafish embryos and the association between *PHF8* mutations and cleft/palate in patients, we next asked whether *Phf8* KO mice are born at sub-Mendelian ratios due to developmental defects or clefting. *Phf8* KO mice were recovered at the predicted Mendelian ratios and survived to adulthood (Supplementary Fig. 3a; Supplementary Table 1), demonstrating that *Phf8* is not required for embryonic development or postnatal survival. Moreover, we failed to detect cleft palate in *Phf8* KO mice (Supplementary Fig. 3c), indicating that loss of *Phf8* on a 129/B6 background is insufficient to cause detectable craniofacial abnormalities.

We were also unable to detect any other gross physiologic abnormalities or differences in the weight of adult *Phf8* KO mice compared to WT controls (Supplementary Fig. 3d), which is in contrast to the growth deficit of cultured *Phf8* mutant cells. However, a closer examination of the brain revealed a subtle yet significant reduction in striatal volume (Supplementary Fig. 4a and b) even though total brain size and architecture appeared normal (Supplementary Fig. 4c and d). We conclude that the proliferation defects of cultured cells are effectively compensated during development and in the adult with the possible exception of the developing striatum. Considering that behavioural abnormalities have been associated with defects of the striatum and *PHF8* has been implicated in intellectual disability, we next evaluated potential behavioural phenotypes in *Phf8* KO mice.

### *Phf8* ^*−/y*^ mice do not show cognitive dysfunction

We subjected *Phf8* KO mice to a battery of behavioural assays to determine whether they mirror the intellectual deficits observed in *PHF8* deficient patients (Supplementary Table 2). Briefly, we utilized the radial arm maze assay as an assessment of working memory and the contextual fear conditioning assay as a measure of learning. Both tests are disrupted in multiple established mouse models of intellectual disability^18^,^19^,^20^,^21^. Surprisingly, neither assay showed significant differences between WT and KO animals (Supplementary Fig. 5a and b), suggesting that loss of *Phf8* in this mouse model does not produce a phenotype consistent with cognitive dysfunction.

### *Phf8* ^*−/y*^ mice are more resilient to stressful stimuli

**Figure 2:**
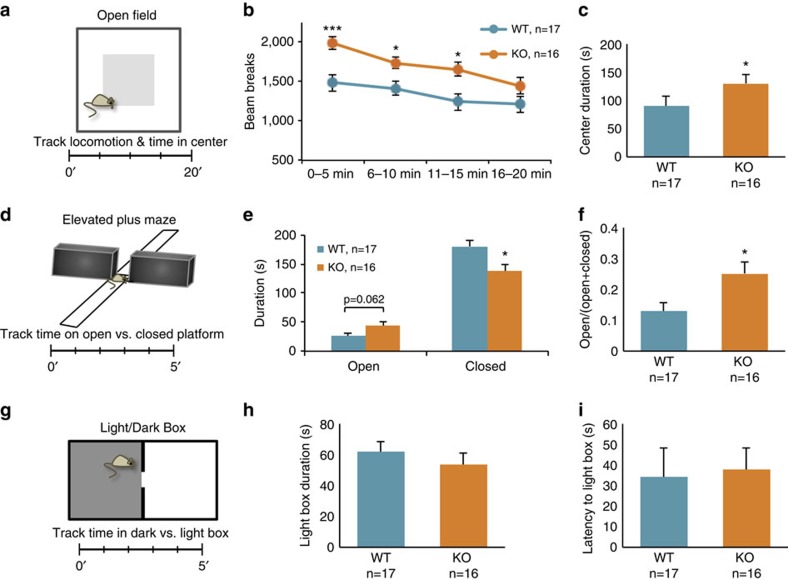
Loss of *Phf8* confers resilience to anxiety. (**a**) Diagram of open field test. Mice are placed in an open, lit chamber and locomotion and time spent in the centre are monitored for 20 min. (**b**) Locomotion as quantified by sensor beam breaks within the open field chamber. (**c**) Time spent in the centre by *Phf8* WT and KO mice. (**d**) Diagram of the elevated plus maze assay. Mice are placed in the centre of an elevated maze containing two open and two enclosed arms; time spent on each arm is monitored for 5 min. (**e**) Quantification of time spent by *Phf8* WT and KO mice on each arm during the elevated plus maze assay. (**f**) Quantification of the fraction of time spent in the open arms over the total time for each genotype. (**g**) Scheme for light/dark box assay. Mice are placed in a container containing an enclosed ‘dark' half and an open ‘light' half. Time spent in either half is measured over a 5-min period. (**h**) Quantification of results from the Light/Dark box assay; the total time spent in the anxiogenic light box is shown. (**i**) Latency to first entry to the light box in the Light/Dark box assay is shown. Error bars=s.e.m. *=*p*<0.05, ^***^=*p*<0.001 (Student's *t*-test two-Tailed).

We next evaluated whether *Phf8* KO mice exhibit aberrant behaviour in response to stress-inducing stimuli. Towards this end, we subjected a cohort of *Phf8* KO animals to an open field test, which measures activity and the amount of time animals spend in the centre or periphery of an open chamber, a readout for anxiety-like behaviour^22^ (Fig. 2a). This assay showed that *Phf8* KO mice are more active than WT controls within the first 15 min of the assessment and spend more time in the centre of the chamber, suggesting reduced anxiety (Fig. 2b,c). We next performed the elevated plus maze test as another measure of anxiety-like behaviour^23^. In this assay, mice are placed at the centre of an elevated four-armed maze with two open and two covered arms; the amount of time a mouse spends in the covered versus the open arms provides a readout of the animal's anxiety (Fig. 2d). This assay revealed that *Phf8* mutant mice spend significantly less time in the closed arms and more time in the open arms, supporting the notion that loss of *Phf8* leads to a decrease in anxiety (Fig. 2e,f). Although we failed to detect a significant difference between *Phf8* KO and WT mice using a third anxiety test, the light-dark box assay^24^ (Fig. 2g–i), our results suggest an unexpected involvement of *Phf8* in conferring resilience to anxiety in a variety of anxiogenic situations.

**Figure 3:**
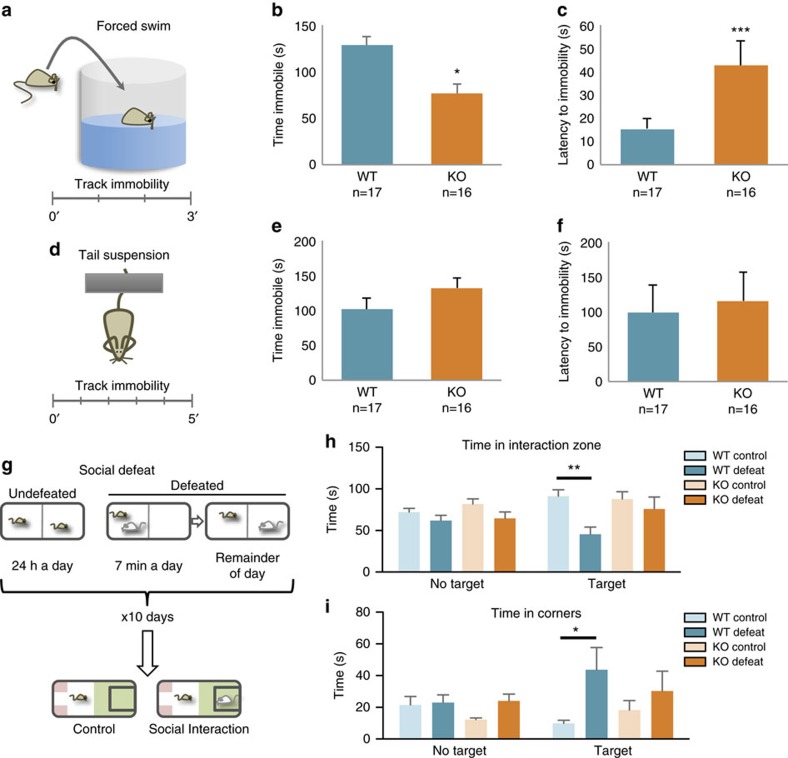
*Phf8* deficient mice are resilient to depression. (**a**) Experimental setup for forced swim assay. Mice are placed in a platform-less beaker filled with water and subsequently monitored for 3 min. (**b**) Time spent immobile in forced swim assay. (**c**) Latency to first immobility in forced swim assay. (**d**) Tail-suspension assay diagram. Mice are taped, suspended by the tail and their immobility is monitored for 5 min. (**e**) Time immobile in a tail suspension assay. (**f**) Latency to first immobility for tail suspension assay (**g**) Experimental design of social defeat assay. Experimental mice are subject to social defeat by exposure to an aggressive mouse over a 10-day training period, then placed with a novel mouse and time spent in the interaction zone (green) versus opposite corners (red) is quantified. (**h**) Time spent in interaction zone for social defeat assay. Control: no social defeat during training phase, No target: no novel mouse in experimental phase, Target: novel mouse in experimental phase. (**i**) Time spent in corners during social defeat assay. WT control no target *n*=10, WT defeat no target *n*=9, KO control no target *n*=10, KO defeat no target *n*=10, WT control target *n*=10, WT defeat target *n*=7, KO control target *n*=10, KO defeat target *n*=9. Error bars=s.e.m. *=*p*<0.05, ^**^=*p*<0.01 (Student's *t*-test, two-tailed).

Anxiety and depression are regulated by overlapping pathways^25^ and both conditions show a significant degree of comorbidity^26^. We therefore probed whether *Phf*8 KO mice also exhibit an anti-depressive phenotype. We first employed a forced swim assay, an acute stress assay which involves placement of the mouse into a water-filled beaker in order to measure immobility time and the latency before the animal first becomes immobile (Fig. 3a). In this assay, latency to immobility directly correlates with an antidepressant response, whereas the amount of time spent immobile inversely correlates with an antidepressant response^27^. We observed that *Phf8* KO mice spend significantly less time immobile than their WT counterparts (Fig. 3b). Moreover, *Phf8* KO animals display a striking increase in latency before the first period of immobility (Fig. 3c). However, *Phf8* KO mice did not show significant differences compared to controls in immobility time in an alternative acute stress assay, the tail suspension test^27^ (Fig. 3d–f). Thus, we subjected *Phf8* KO mice to a chronic social defeat stress paradigm, which represents an independent, robust measurement of depressive behaviour^28^. In brief, experimental mice are subjected to social defeat by exposure to an aggressive mouse over a 10-day training period. Subsequently, experimental mice are placed into a new cage containing a novel mouse (Fig. 3g). The amount of time the experimental mouse spends interacting with or avoiding the novel mouse serves as a readout for social avoidance, which has been correlated with several other depression-related behavioural abnormalities^28^,^29^. Defeated WT mice showed the expected social avoidance behaviour, as determined by less time spent in the interaction zone and more time spent in the corners when compared to an undefeated WT animal (Fig. 3h,i). In contrast, *Phf8* KO mice did not display any signs of avoidance behaviour following social defeat, spending a comparable amount of time in the interaction zone and corners as undefeated *Phf8* KO and WT animals. Altogether, these data indicate that *Phf8* KO mice have a remarkable resiliency to anxiety- and depression-like behaviours using several independent stress-inducing paradigms, thus uncovering a biological role for PHF8 not previously associated with this histone demethylase.

### Molecular defects in the prefrontal cortex of *Phf8* ^*−/y*^ mice

**Figure 4:**
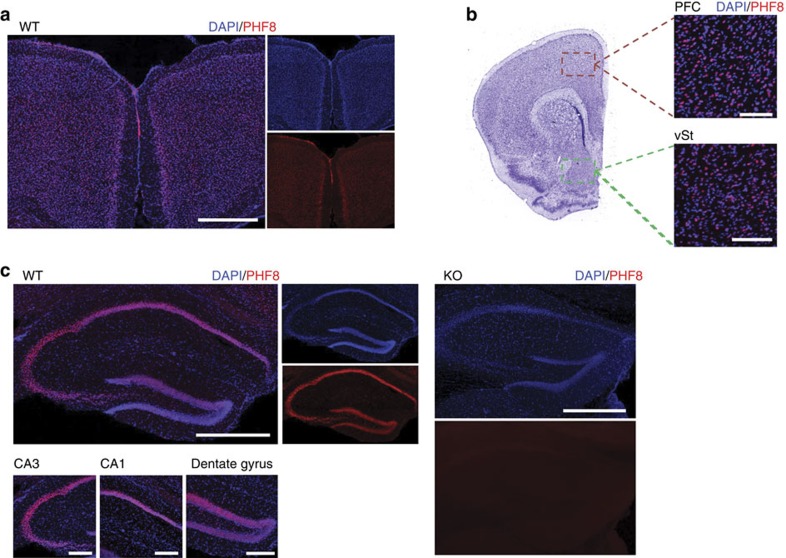
Characterization of Phf8 expression in brain regions relevant to anxiety and depression. (**a**) PHF8 expression (red) in the neocortex. Note that PHF8 is most strongly expressed in layer V, although it is also detectable in layers II/III. Scale bar, 500 μm. (**b**) Expression of PHF8 in the prefrontal cortex (PFC) and ventral striatum (vSt) Scale bar, 100 μm. (**c**) PHF8 expression in the dorsal hippocampus of WT (left) mice, no signal is detected in the KO control (right). PHF8 appears broadly expressed throughout the hippocampus. Top scale bar (whole hippocampus), 500 μm. Bottom scale bar (CA3, CA1, DG), 200 μm.

**Figure 5:**
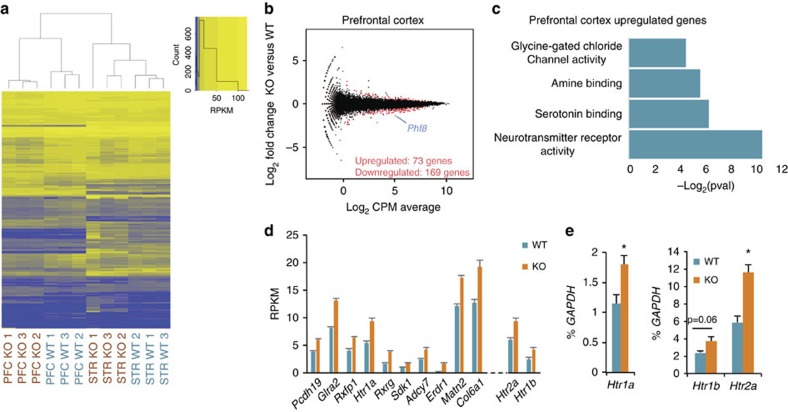
Loss of *Phf8* in the prefrontal cortex causes misregulation of serotonin receptors. (**a**) Unbiased hierarchical clustering and heatmap for differentially expressed genes between *Phf8* WT and KO prefrontal cortex and ventral striatum. (**b**) Mean Average plots comparing *Phf8* WT and KO RNA-seq data from prefrontal cortex. Differentially expressed genes (*p*<0.05, FDR<0.05) between WT and KO for each sample set are shown in red. (**c**) Gene ontology (GO) molecular function analysis (GO database released 2016-09-24) of the differentially expressed (upregulated and downregulated) gene sets from the prefrontal cortex (1.4 fold cutoff, FDR<0.05). The negative log_2_ of the Bonferroni corrected *p* value is displayed along the *x*-axis for all categories for which *p*<0.05. Enrichments for each category: Neurotransmitter Receptor Activity 6/93, Serotonin Binding 3/11, Amine Binding 3/13, Glycine Gated Channel Activity 2/2. (**d**) RNA-seq expression data of the top 10 most significantly differentially expressed genes in the prefrontal cortex ranked in order of descending *p* value, note the presence of the *Htr1a* serotonin receptor. Also shown (out of rank order) are the serotonin receptors *Htr2a* and *Htr1b*. (**e**) RT-qPCR analysis confirms upregulation of *Htr1a, Htr1b* and *Htr2a* expression in the prefrontal cortices of an independent set of mice (WT: *n*=3, KO: *n*=4). Error bars=s.e.m. *=*p*<0.05 (Student's *t*-test, two-tailed).

To understand the molecular and cellular basis for the observed behavioural phenotype, we first determined *Phf8* expression patterns in the brain. In adult mice, PHF8 protein is present across forebrain neurons of the entire neocortex, the hippocampus and the ventral striatum (Fig. 4a–c). Considering that altered gene expression in each of these structures has previously been linked to resiliency to depression^30^,^31^,^32^, we performed RNA-sequencing on the ventral striatum and prefrontal cortex of *Phf8* KO and WT animals. Unlike global expression analysis of ESC and NPC samples, which failed to show a separation of *Phf8* KO and WT samples (Supplementary Fig. 2), we found that the transcriptomes of prefrontal cortex and ventral striatum samples cluster by genotype using unsupervised hierarchical clustering (Fig. 5a). A comparison of differentially expressed genes between *Phf8* KO and control samples further revealed 73 upregulated and 169 downregulated genes within the prefrontal cortex, and 79 upregulated and 18 downregulated genes within the ventral striatum with a FDR<0.05 (Fig. 5b; Supplementary Fig. 6a).

To assess whether any particular biological processes are associated with the dysregulated genes in *Phf8* KO cells, we next applied gene ontology (GO) analysis on the up- and downregulated gene sets (GOTERM_MF; >1.4 fold change; FDR<0.05; Fig. 5c). We found that ‘Neurotransmitter Receptor activity', ‘Serotonin Binding' and ‘Amine Binding' were the top-enriched categories when considering upregulated genes in the prefrontal cortex (Fig. 5c), which is in agreement with the notion that serotonin signalling plays an important role in modulating anxiety and depression^33^. GO analysis of differentially expressed genes from the ventral striatum revealed categories related to synaptic transmission (Supplementary Fig. 6b). However, a number of top differentially expressed genes between *Phf8* KO and WT striatal samples, notably *Oxt* and *Avp*, could not be validated by qRT-PCR using additional mice, suggesting that some of these initially observed differences were the result of contamination from the hypothalamus (Supplementary Fig. 6c and d). Given the cleaner expression data of the prefrontal cortex and the well-established link between serotonin signalling and depression, we focused on the molecular mechanisms by which *Phf8* may perturb serotonin signalling in the prefrontal cortex in the remainder of this study.

### PHF8 directly regulates serotonin receptors in the neocortex

Inspection of differentially expressed genes in the prefrontal cortex of *Phf8* KO mice revealed upregulation of multiple serotonin receptors including *Htr1b, Htr2a* and *Htr1a,* which have previously been linked to anxiety and depressive phenotypes in rodents^34^,^35^,^36^,^37^ (Fig. 5d). We confirmed upregulation of this serotonin receptor family in an additional cohort of *Phf8* KO mice (*n*=3 for WT and *n*=4 for KO) using RT-qPCR (Fig. 5e), indicating that lack of *Phf8* leads to a subtle yet significant misregulation of serotonin signalling in the prefrontal cortex; we note that other candidates identified by RNA-Seq exhibited more variable expression in these samples (Supplementary Fig. 7a and b).

Given that PHF8 has specificity for repressive histone modifications, direct targets are expected to be downregulated rather than upregulated in the absence of PHF8. We therefore considered the possibility that PHF8 may target a repressor of serotonin receptor expression, thus explaining the observed transcriptional upregulation of *Htr* genes in the absence of PHF8. Towards this end, we surveyed the expression levels of transcription factors that are predicted or known to bind to the promoter and enhancer elements of the *Htr1a* locus based on previous studies^38^. However, none of these candidate factors were differentially expressed between *Phf8* KO and control mice (Supplementary Fig. 8), suggesting that *Htr1a* upregulation is unlikely to be the consequence of an indirect mechanism involving these repressors.

**Figure 6:**
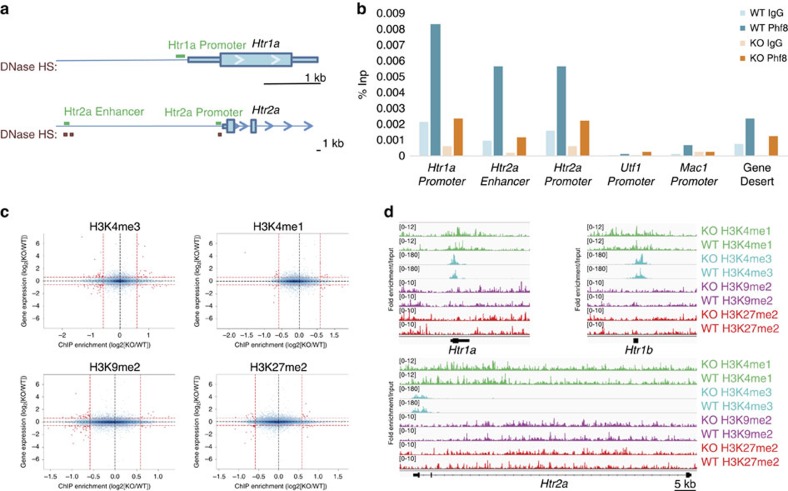
PHF8 directly regulates the serotonin receptors *Htr1a* and *Htr2a.* (**a**) ChIP primers (green bars) selected in promoter and putative enhancers of *Htr1a,* and *Htr2a*. Promoter-specific primers were designed in conserved regions within the first 300 bp of the TSS. Enhancer-specific primers were placed at conserved cerebral DNase hypersensitivity sites (HSs) (Red boxes; ENCODE/University of Washington) upstream of the TSS. For *Htr2a* a putative long range enhancer (∼40 kb) was selected, with *Htr2a* being the closest gene to this site and the next nearest gene being >100 kb away. (**b**) ChIP-qPCR on the neocortex with primers located in conserved regions either ∼200 bp upstream of the TSS (Promoter) or within a putative enhancer for the serotonin receptors. PHF8 occupancy is detected at the *Htr1a* and *Htr2a* loci but not at the promoter of *Utf1*, *Mac1* nor within a gene desert. (**c**) Correlation between differential gene expression (*y*-axis; log_2_[KO/WT]) by RNA-seq and differential histone mark deposition (*x*-axis; log_2_[KO/WT]) at the TSS±3 kb when comparing WT and KO in neocortical neurons. (**d**) Input normalized reads show the fold enrichment of H3K4me1, H3K4me3, H3K9me2 and H3K27me2 at the serotonin receptors *Htr1a, Htr1b* and *Htr2a*.

To explore whether PHF8 may be directly recruited to serotonin receptor loci to suppress their expression, we performed chromatin immunoprecipitation (ChIP) followed by quantitative PCR on the neocortices of *Phf8* KO and WT mice. We observed significant enrichment at the promoters of both *Htr1a* and *Htr2a* as well as at a putative enhancer for *Htr2a*, as defined by an upstream conserved cerebral DNaseI hypersensitivity site (Fig. 6a,b; Supplementary Fig. 9). No significant enrichment of PHF8 was observed within a gene desert, at the promoter regions of the pluripotency and lineage-specific genes *Utf1* and *Mac1* nor at the *Htr* loci of *Phf8* KO mice (Fig. 6b), confirming the specificity of the PHF8 ChIP. Altogether, these data provide evidence that PHF8 is directly recruited to at least these two serotonin receptor loci and likely represses their expression.

To determine whether *Phf8* loss affects histone methylation patterns in the brain, we performed ChIP combined with sequencing (ChIP-Seq) on neuronal chromatin from FACS-sorted NeuN^+^ nuclei^39^ extracted from WT and KO neocortices (*n*=2). Specifically, we analysed H3K9me2 and H3K27me2, which are repressive modifications targeted by PHF8, as well as H3K4me3 and H3K4me1, which are activating marks of promoters and enhancers, respectively. Surprisingly, we did not observe any correlation between differential enrichment for any of these histone modifications and gene expression changes in WT versus KO samples when analysing regions within 3 kb of transcription start sites (TSSs) (Fig. 6c), where PHF8 is expected to bind^11^,^13^,^15^. Likewise, we failed to detect differences in the distribution of these marks when extending our analysis to gene bodies for H3K9me2 and to enhancers^40^ for H3K27me2 and H3K4me1 of differentially expressed genes (Supplementary Fig. 10a,b). GO analyses of promoters (TSS±3 kb), enhancers, and gene bodies that showed differential signals for each of these marks regardless of expression status also did not reveal significant enrichment for any molecular pathways (GOTERM_MF; loci enriched/depleted >2 fold; FDR<0.05). Moreover, analysis of the promoters and gene bodies for *Htr1a, Htr2a* and *Htr1b* did not reveal significant differential enrichment for any of the four analysed histone methyl-marks (Fig. 6d). Altogether, these analyses suggest that PHF8's demethylase function is compensated in the neocortex of adult KO mice and thus cannot explain the observed transcription changes we detected.

## Discussion

We have shown here that targeted deletion of PHF8's catalytic domain is dispensable for normal mouse development and survival to adulthood. These results are surprising as they differ from previous data in zebrafish, which reported craniofacial and brain abnormalities following morpholino-based knockdown of *phf8*. We surmise that differences in our strategies to deplete *Phf8* may account for some of the distinct phenotypes. For example, it is possible that morpholinos targeting *Phf8* also recognized homologous family members, resulting in an exacerbated phenotype. Alternatively, distinct phenotypes between zebrafish and mouse might be caused by species-specific differences or differences in genetic background.

Despite the lack of an obvious phenotype during development and postnatal life of *Phf8* deficient mice, primary cultures established from KO animals (ESCs, NPCs and MEFs) showed a subtle proliferation defect, confirming and extending previous studies using shRNA-mediated knockdown of *Phf8* in transformed cell lines^11^,^13^,^14^,^15^. However, considering that *Phf8* KO mice do not show any apparent growth deficits, we hypothesize that the proliferation defects of cultured cells are effectively compensated for during development and in the adult.

While *PHF8* mutations have previously been associated with intellectual disability and cleft lip/palate in patients, we failed to obtain evidence for either cognitive or craniofacial abnormalities in *Phf8*-deficient mice maintained on a 129/B6 background. It is worth mentioning that the cleft lip/palate phenotype is not fully penetrant in patients carrying *PHF8* mutations. Moreover, the degree of intellectual disability varies greatly among *PHF8*-deficient patients^6^,^7^,^8^, suggesting that other environmental or genetic factors likely contribute to the manifestation of the disease. It should be informative to backcross *Phf8*-mutant mice onto different genetic backgrounds to test this hypothesis.

Notably, *Phf8* deficient animals display resilience to anxiety- and depression-related behavioural abnormalities, which uncovers a novel biological role for PHF8 not previously associated with this histone demethylase. In agreement with this observation, we find that PHF8 is abundantly expressed in structures of the forebrain that are known to contribute to resilience toward anxiety and depression including the prefrontal cortex, ventral striatum and hippocampus^34^,^35^,^36^,^37^,^41^. Critically, our RNA-Seq and ChIP-qPCR data provide a direct mechanistic link between the lack of PHF8 and elevated expression of serotonin receptors including 5-HT1A, 5-HT1B and 5-HT2A. Multiple lines of evidence suggest that increased expression of these serotonin receptors is indeed responsible for the observed resilience phenotype of *Phf8* KO mice. First, SSRIs, which enhance serotonin signalling by inhibiting its reuptake, are among the most commonly prescribed antidepressants and antianxiety medications^42^. Second, patients with depression exhibit a reduced binding potential for the 5-HT1A receptor in the prefrontal cortex and hippocampus and for the 5-HT2A receptor in the prefrontal cortex^43^. Third, postmortem tissue from suicide victims shows reduced *HTR1B* mRNA levels within prefrontal cortex and hippocampus^44^. Fourth, mice deficient for *Htr1a* display increased anxiety- and depression-like behaviours^34^, whereas treatment of rodents with 5-HT1A, 5-HT1B or 5-HT2A agonists have similar anti-anxiety effects^35^,^36^,^37^,^45^.

PHF8's apparent repressive role in regulating serotonin receptor expression may seem counter-intuitive considering its reported function as an activator of transcription. Moreover, our ChIP-seq data did not point towards aberrations of particular histone methylation patterns as a consequence of *Phf8* loss. While we cannot exclude that *Phf8* KO mice exhibit differences in histone methylation in a rare neuronal subpopulation that our ChIP-seq approach was unable to detect, this possibility is unlikely to explain the specific effect *Phf8* loss has on serotonin receptor expression. We note that the repressive effect of PHF8 on serotonin receptor expression in our system is consistent with previous studies, which reported an increase in the expression levels of a subset of direct PHF8 targets on knockdown^13^,^17^. Furthermore, the closely related histone demethylase PHF2, which shares PHF8's specificity toward H3K9me2 (ref. 46), functions as a repressor in some contexts by recruiting the H3K9 methyltransferase Suv39h1 (ref. 47). In addition, a demethylase-independent role in transcriptional regulation has been reported for PHF8 through direct interaction with the core transcriptional machinery^13^. We thus hypothesize that PHF8 functions similarly by recruiting as yet unidentified co-repressors to a subset of target loci including *Htr1a* and *Htr2a*. It will be critical to identify cofactors of PHF8 and their association to specific targets in neuronal cells in the future to better understand PHF8's demethylase-independent function and apparent dual role in gene repression and activation.

Depression and anxiety disorders represent the most common mental disorders in the developed world with respect to years lost to disability^48^, yet patient response to available treatments is often incomplete^49^. A more complete understanding of the mechanisms underlying anxiety and depression is therefore critical to develop more effective treatments. Our results raise the possibility that PHF8 is also involved in controlling anxiety and depression in humans and may thus represent a novel drug target. Preliminary analysis of published ChIP-Seq data^50^ indeed show that *HTR1A* is a direct target of PHF8 in human embryonic stem cells, suggesting conserved mechanisms. While caution is certainly warranted considering *PHF8's* association with intellectual disability, it is plausible that inhibition of PHF8 in the adult modulates anxiety/depression without affecting intellectual capacity.

## Methods

### Generation of *Phf8* knockout mice

A targeting construct containing *Phf8* exons 7 and 8 flanked by *loxP* sites and a neomycin resistance cassette was electroporated into V6.5 ESCs. Electroporated ESCs were selected with G418, picked, and clonally expanded. Integration of the targeting construct was confirmed by Southern blot. Positive clones were electroporated again with a CMV-Cre expression vector, picked, and clonally expanded again. Loop out of exon's 7 & 8 was confirmed by Southern blot and positive clones were injected into E3.5 BDF1 blastocyts and transferred into the uteri of surrogate Swiss Webster mice. The knockout allele was maintained on a mixed C57B6/129SvJae background. All animal experiments were approved by the IACUC committee and conform to the regulatory standards.

### Western blot analysis

Whole cell extract or, for histone analysis, nuclear extract was run on either 10 or 15% SDS–polyacrylamide gel electrophoresis (SDS–PAGE) gels at 150 V until separated and transferred at 4 °C for either 1 h at 80 V (histones) or overnight at 20 V (Phf8) onto a PVDF membrane. Membranes were blocked in 5% milk PBS-T (1 × PBS, 0.1% Tween-20) for 30 min at RT then incubated with primary antibodies for 1 h in block at RT. Membranes were washed in PBS-T, and incubated in the appropriate horseradish-peroxidase-conjugated secondary for 45 min at RT. Membranes were then washed again and visualized using ECL reagents (Pierce). Primary antibodys: Phf8 Abcam ab36068 1:400, H3 Abcam ab1791 1:5,000, H3K9me1 Abcam ab8896 1:500, H3K9me2 Abcam ab1220 1:200, H3K9me3 Abcam ab8898 1:1,000, H3K27me2 Millipore 07-452 1:2,000, H4 Abcam ab17036 1:1,000, H4K20me1 Abcam ab9051 1:1,000, beta Actin-HRP Abcam ab20272 1:8,000. All uncropped blots can be found in Supplementary Fig. 11.

### Southern blot analysis

20 μg of genomic DNA collected from ESCs was digested overnight at 37 °C with 500 U μl^−1^ of StuI (New England Biolabs), then electophoresed on a 0.8% agarose gel at 70 V until separated (6–8 h). The gel was depurinated in 0.5 N HCl for 25 min at RT and quenched in transfer buffer (1.5 M NaCl, 0.5 M NaOH) for 45 min at RT. Samples were transferred to a Hybond-XL membrane (GE Healthcare) via a downward transfer for 14–16 h. The membrane was washed for 15 min at RT in 2 × SSC then blocked for an hour at 65 °C in Hyb buffer (0.5 M NaP_i_, 7% SDS, 1 mM EDTA). Probes (See Supplementary Table 3 for probe primer sequences) were labelled with Prime-IT II Random Primer Labeling Kit (Agilent) and added to membranes in 30 ml of fresh Hyb buffer, hybridization was run overnight. Membranes were washed 2 × with wash buffer (40 mM NaP_i_, 0.1% SDS) for 15 min and 30 min before exposure.

### Proliferation and neurosphere formation assays

For MEFs and ESCs equal numbers of cells were plated into triplicate wells of a six well plate, 20,000 cells per well for MEFs and 5,000 cells per well for ESCs. Wells containing ESCs were gelatinized with 0.2% gelatin to support attachment. Cells were stained with trypan blue and trypan blue negative cells were counted every 3 days.

For neurosphere formation assays, undifferentiated NPCs (CD24^−^EGFR^+^) were sorted and plated at low density to avoid clumping, 10 cells per μl, in low attachment 6-well or 24-well plates. New neurospheres were allowed to grow for 1 week and imaged in brightfield. Sphere diameters were measured in NIS Elements software (Nikon), the shortest possible diameter was recorded for all spheres. Statistical significance was calculated with a 2-tailed student's *t*-test.

### Behavioural analyses

Open field locomotion was monitored in a 40 × 40 × 40 cm^3^ clear plastic arena using a photocell-beam-based detecting system (OmniTech Electronics. Inc). Animals were introduced into the corner of the chamber and allowed free exploration for 20 min individually under standard lighting conditions. Beam breaks were recorded every 5 min as was the total distance travelled and the duration in the centre.

The elevated plus maze (Med Associates Inc) contained a 6 × 6 cm centre square, two open and two closed arms at 35 × 6 cm^2^ each. Closed arms were enclosed with black walls measuring 20 cm in height. Mice were placed in the centre square facing one of the closed arms to begin the assessment. Time spent in each arm was scored by the EthoVision video tracking system (Noldus, Wageningen, The Netherlands).

The light/dark box test was performed in an open field with a black box insert measuring 20 cm L × 20 cm W × 40 cm H, which divided the arena into a light and dark component connected by a small hole. Mice were introduced into the dark chamber and allowed free exploration for 10 min. Duration spent in each chamber was tracked during this time.

For the forced swim assay, animals were placed in a 4L Pyrex beaker 13 cm diameter and 24 cm high filled with 17 cm of 22 °C water. Activity of the animals was tracked for 5 min and latency to first freeze, as well as time spent immobile was assessed by EthoVision.

In the tail suspension test, mice were suspended by the tail with duct tape and videotaped for 5 min. Latency to first freezing and time spent immobile was evaluated with EthoVision software, animals displaying tail climbing behaviour were excluded from the analysis.

In the contextual fear conditioning assay, mice were placed in a fear conditioning chamber measuring 30.5 × 24.1 × 21 cm^3^ (Med Associates, Inc.) for a 7 min training session. During this time three shocks were delivered and the percentage of time freezing before the first shock (baseline) and after each shock was measured. After 24 h, mice were returned to the chamber for a 3 min retrieval testing and percentage of time freezing was measured.

For the radial arm maze test, mice were placed in the centre of an 8 armed apparatus, each arm measuring 5 × 50 cm^2^ with 30 cm high walls. Mice were assessed for four consecutive days and each day the test was continued until all eight arms had been visited at least once. A mistake was defined as a repeated entry into an already visited arm. Total mistakes during a session were measured.

For the chronic social defeat stress assay, mice were subjected to 7 min social defeat by a novel CD1 aggressor, after which time they were housed together across a plexiglass divider allowing for sensory contact for the rest of the day. This process was repeated daily for 10 days. Control mice were housed in cages separated from other control mice and were rotated to a different cage daily. After 24 h of the last manipulation, experimental and control mice were placed in an arena 44 × 44 cm^2^ containing a novel CD1 mouse within a Plexiglas/wire mesh enclosure measuring 10 × 6 cm against one wall of the arena. Time spent in the 14 × 26 cm^2^ interaction zone around the enclosure and time spent in the opposite corners was quantified using EthoVision software.

### Immunofluorescence analysis

Animals were anesthetized with avertin before transcardial perfusion with 20 ml cold 4% PFA in PBS. Brains were dissected and post-fixed 14–16 h in 4% PFA PBS following which they were cryoprotected in 30% sucrose PBS. Cryoprotected brains were frozen in OCT on dry ice and 30 micron floating sections were cut using a Leica cryostat. Floating sections were washed in PBS 0.3% triton 15 min at RT, then blocked in 0.3% triton, 10% normal donkey serum and PBS for 2 h at RT. Sections were incubated with primary antibodies overnight at 4 °C in PBS 0.3% triton. Sections were washed 3 × 15 min in PBS and incubated with Alexa488, Alexa546 or Alexa647 conjugated secondary antibodies in PBS for 2 h at RT. Sections were washed 3 × 15 min in PBS with DAPI added to the second wash and mounted in Prolong Gold Antifade (Life Technologies) for imaging. Primary antibodies: Phf8 Abcam ab191386.

### Data analysis & statistics

A student's two-tailed *t*-test was used to determine statistical significance. Samples were considered significantly different if *p*<0.05. Normality was tested and confirmed with the D'Agostino & Pearson Normality test. Sample size for behavioural experiments was chosen based on animals used to achieve sufficient statistical power in previously published studies. Behavioural experiments were not blinded with the exception of the chronic social defeat stress assay. Additional statistical methods relevant to the analysis of deep sequencing is described in the following sections.

### Bioinformatic analyses of RNA-seq data

Data was aligned with the splace-aware alignment program STAR (http://bioinformatics.oxfordjournals.org/content/early/2012/10/25/bioinformatics.bts635) to map the sequencing reads to a mouse reference genome (assembly mm10/GRCm38). Gene expression counts were calculated using the program HTSeq (http://www-huber.embl.de/users/anders/HTSeq/doc/overview.html) based on a current Ensembl annotation file for mm10/GRCm38 (release 75). We then used the R package ‘edgeR' (https://bioconductor.org/packages/release/bioc/html/edgeR.html) to make differential gene expression calls from these counts according to the following criteria: gene expression was considered to be UP-regulated if log2 FC>+1 or DOWN-regulated if the log2 FC<−1 (FC=fold-change of CPM) with respect to the conditions being compared at an arbitrary false discovery rate (FDR) value<0.05. GO analysis was performed using DAVID (https://david.ncifcrf.gov/) for ‘GO Molecular Function.'

### Hierarchical clustering

Heatmaps/dendrograms of the 12 samples were constructed using RPKM expression values of all genes for PFC and STR samples which registered an FDR<0.05 for differential expression. Heatmaps were created using the ‘heatmap.2 function from the gplots package for R′ (http://www.inside-r.org/packages/cran/gplots/docs/heatmap.2) in conjunction with the hierarchical clustering option ‘ward.D2.'

### RNA preparation for sequencing and RT-qPCR

RNA from ESCs, NPCs, the prefrontal cortex or the ventral striatum was purified using an RNeasy kit (Qiagen) and including the optional DNase digest step. For sequencing, quality of RNA was assessed using an Agilent RNA 6000 Nano kit. For library preparation, Illumina TruSeq RNA Sample Prep Kit was used with 1 μg of sample RNA. Library quality was again assessed with the BioAnalyzer Agilent High Sensitivity DNA kit. Bar-coded libraries were then pooled at equimolar concentration and sequenced on an Illumina HiSeq 2000.

For RT-PCR, 500 ng of purified RNA was used for an RT reaction with the Transcriptor First strand cDNA synthesis kit (Roche). cDNA reactions were heat inactivated, diluted 1:10–1:50 and analysed by qPCR on a Roche LightCycler 480 with a Brilliant III Ultra-Fast SYBR Green kit (Agilent). See Supplementary Table 3 for primer sequences.

### Chromatin immunoprecipitation

For PHF8 ChIP-qPCR experiments, the cortex was dissected from adult mice (∼2 months old) homogenized to a single-cell suspension using a dounce homogenizer in ice cold PBS. The suspension was filtered and treated with 1.5 mM EGS (ethylene glycolbis(succinimidylsuccinate)) for 25 min at RT in PBS, crosslinking was continued with the addition of 1% formaldehyde for 10 min at RT. Crosslinking was quenched by adding 0.125 M glycine 10 min RT. Samples were washed with PBS and lysed in 50 mM Tris–HCl pH 8.0, 1% SDS, 4 mM EDTA with protease inhibitor cocktail (Roche complete mini) on ice for 20 min. Lysed samples were sonnicated 10 min total sonication time, in a bioruptor with pulses of 30 seconds on, 30 seconds off then cleared by spinning at max in a tabletop centrifuge for 10 min at 4 °C. Samples were diluted 5 × with dilution buffer (165 mM NaCl, 0.01% SDS, 1.1% Triton-X, 1.2 mM EDTA, 16.7 mM Tris–HCl pH8.0). Samples were precleared with 20 μl streptavidin-agarose beads for 2 h at 4 °C. Around 10% was removed for input and the remainder incubated with 5 μg of primary antibody (Phf8 Abcam ab36068 lot# GR176758 or Rb IgG Abcam) overnight at 4 °C. 8ul of protein-G Dynabeads (Life Technologies) were added to each sample and incubated 2 h at 4 °C. Beads were collected on a magnet and washed 1 × with dilution buffer, 2 × with low salt buffer (150 mM NaCl, 0.5% Sodium deoxycholate, 0.1% SDS, 1% IGEPAL, 1 mM EDTA, 50 mM Tris–HCl pH 8.0), 4 × with high salt buffer (500 mM NaCl, 0.5% Sodium deoxycholate, 0.1% SDS, 1% IGEPAL, 1 mM EDTA, 50 mM Tris–HCl pH 8.0), and twice with TE. All wash buffers contained complete mini protease inhibitor (Roche). Samples were eluted with two rounds of constant vortexing for 10 min in 50ul elution buffer (1% SDS, 100 mM sodium bicarbonate) then brought to a final concentration of 0.3 M NaCl and incubated overnight at 65 °C. Samples were brought to final concentration of 50 mM Tris–HCl pH 6.8, 10 mM EDTA and treated with proteinase K for 1 h at 55 °C then purified with a PCR purification kit (Qiagen). Samples were eluted in water and diluted 1:3 for analysis in qPCR with a Brilliant III Ultra-Fast SYBR Green kit (Agilent) on a Roche LightCycler 480. See Supplementary Table 3 for primer sequences.

ChIP-seq on neuronal chromatin was performed as described previously^39^. Briefly, neocortices were isolated from 2 WT & 2 KO littermates and pooled before nuclei isolation by ultracentrifugation through a sucrose gradient. Neuronal nuclei were purified by immunotagging with a FITC conjugated NeuN antibody before FACS-sorting. Sorted nuclei were MNase digested and ChIP was performed with anti-H3K4me1 (Abcam ab8895), H3K4me3 (Millipore 17-614), H3K27me2 (Millipore 07-452) and H3K9me2 (Abcam ab1220). ChIP libraries were prepared with Illumina TruSeq ChIP Library Prep Kit and sequenced on an Illumina HiSeq 2000.

### Bioinformatic analyses of ChIP-seq data

Sequencing was performed on an Illumina HiSeq 2500 instrument, resulting in approximately 30 million paired-end 50 bp reads per sample on average. Reads were aligned against the mm10 mouse genome build using BWA (ref. 52). For analysis of enhancer regions, alignments against the mm9 build were used. All alignments were filtered for uniquely mapped reads and alignment duplicates were removed. Input-normalized coverage tracks were generated using deepTools version 2.0.1 (ref. 51).

To analyse the epigenetic consequences of the Phf8 knock-out, input-normalized coverages for the marks of interest (H3K4me1, H3K4me3, H3K9me2, H3K27me2) were compared over all transcriptional start sites (TSS)±3 Kb windows and gene bodies. Phf8-dependent changes in mark deposition were further compared against changes in gene expression in the knockout.

### Data availability

RNA-seq and ChIP-seq data have been deposited in the Gene Expression Omnibus database under accession code GSE90020. The authors declare that all data supporting the findings of this study are available within the article and its supplementary information files or from the corresponding author on reasonable request.

## Additional information

**How to cite this article:** Walsh, R. M. *et al*. *Phf8* loss confers resistance to depression-like and anxiety-like behaviors in mice. *Nat. Commun.* **8,** 15142 doi: 10.1038/ncomms15142 (2017).

**Publisher's note:** Springer Nature remains neutral with regard to jurisdictional claims in published maps and institutional affiliations.

## Supplementary Material

Supplementary Information Supplementary Figures, Supplementary Tables.
