# Supplementary material for: Phf8 loss confers resistance to depression-like and anxiety-like behaviors in mice
Source: Nat Commun. 2017 May 9;8:15142. doi: 10.1038/ncomms15142 (PMC5436068; doi:10.1038/ncomms15142)
Supplement: Supplementary Information — Supplementary Figures, Supplementary Tables. [file ncomms15142-s1.pdf]

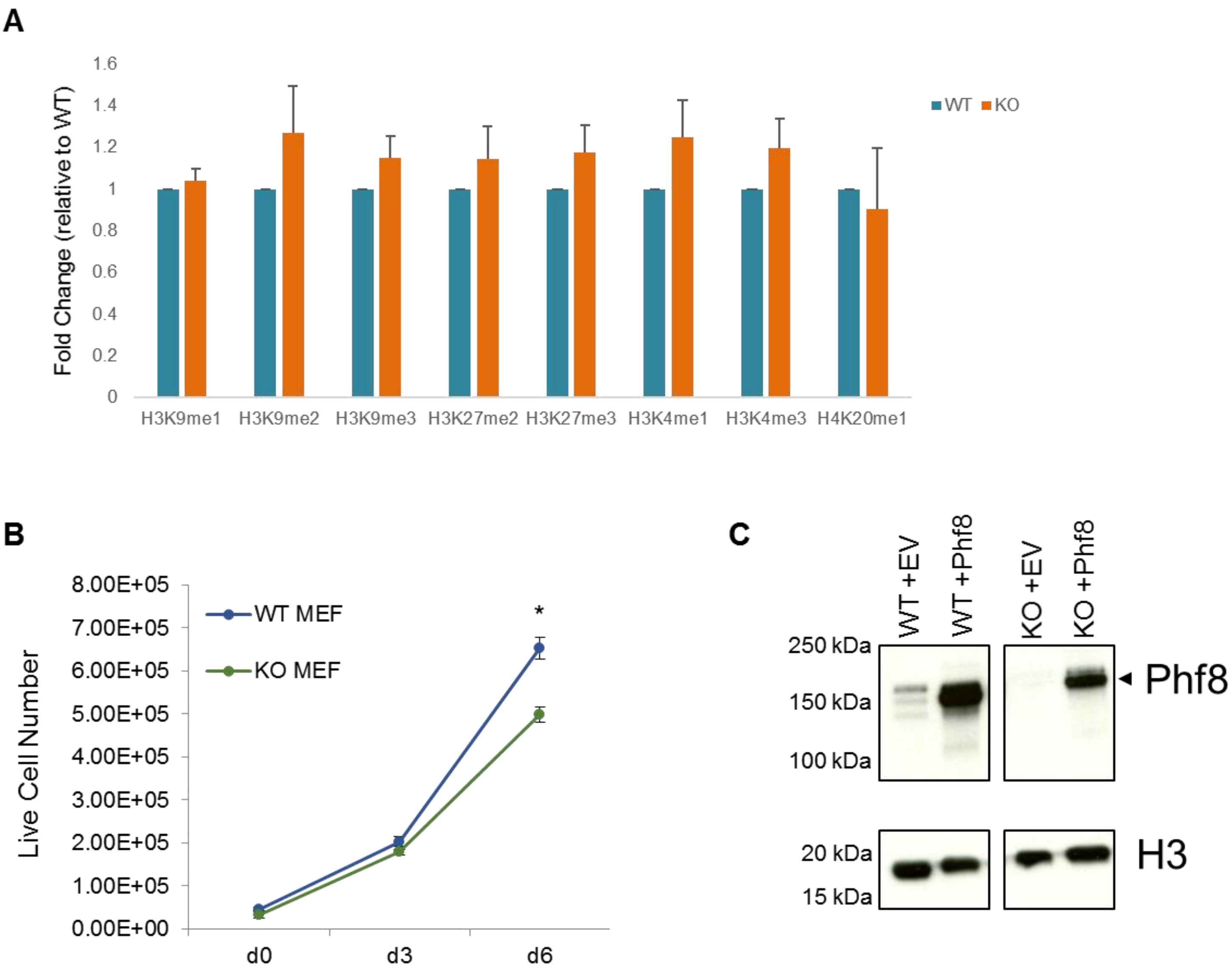

Supplementary Figure 1 Growth curve of *Phf8* KO MEFs. (A) Quantification of Western blot analysis displayed in Figure 1E. Ratio of each H3-normalized signal over WT is displayed. No significant differences are observed. WT n=3, KO n=3. **(B)** Growth curve for triplicate *Phf8* WT and KO MEFs; shown is the average of 3 independent experiments performed in triplicate. **(C)** Western blot analysis pertaining to Figure 1F, showing expression of Phf8 in WT and KO ESCs infected with either empty vector (EV) or full-length Phf8 cDNA (Phf8) lentiviral vectors. Error bars=s.e.m. *=$p<0.05$ (Student's T-test, 2-tailed).

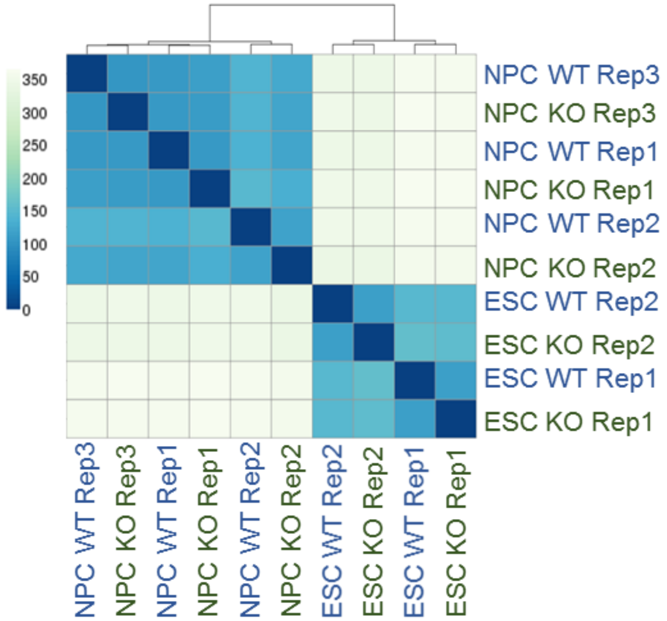

Supplementary Figure 2 RNA-seq identifies subtle defects related to cell cycle in *Phf8* KO ESCs and NPCs. Unbiased hierarchical clustering of *Phf8* WT and KO ESCs and NPCs.

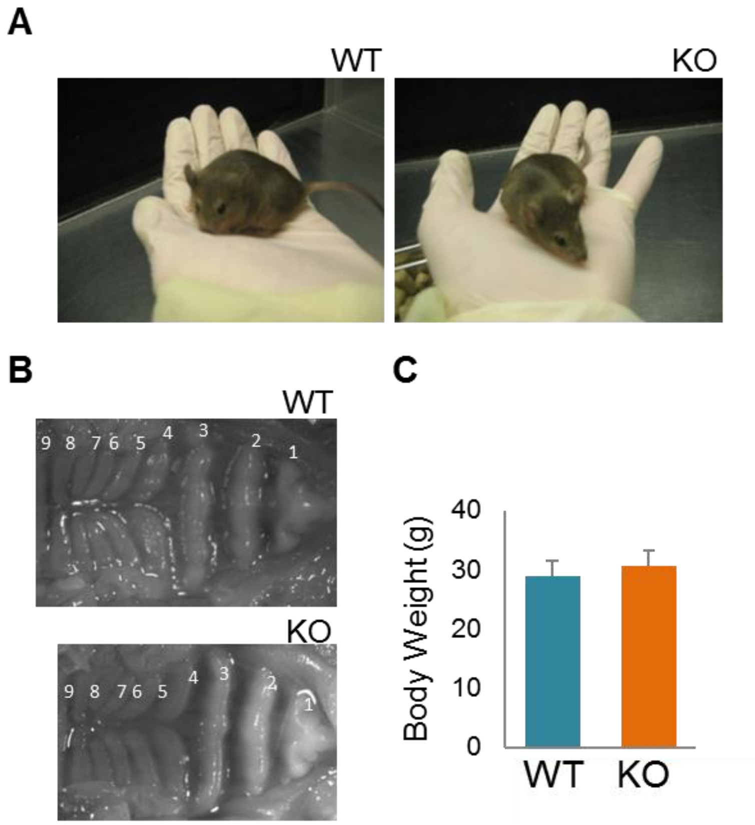

Supplementary Figure 3 *Phf8* KO mice do not exhibit gross physiological defects. **(A)** *Phf8* WT (left) and KO (right) mice are phenotypically indistinguishable. **(B)** Representative images of the palates from *Phf8* WT (n=3) and KO (n=5) mice. Rugae are numbered 1-9, none are absent. **(C)** No difference in body weight seen between adult (2 month old) *Phf8* WT and KO (n=3) mice. Error bars=s.e.m. No significant differences detected (Student's T-test, 2 tailed).

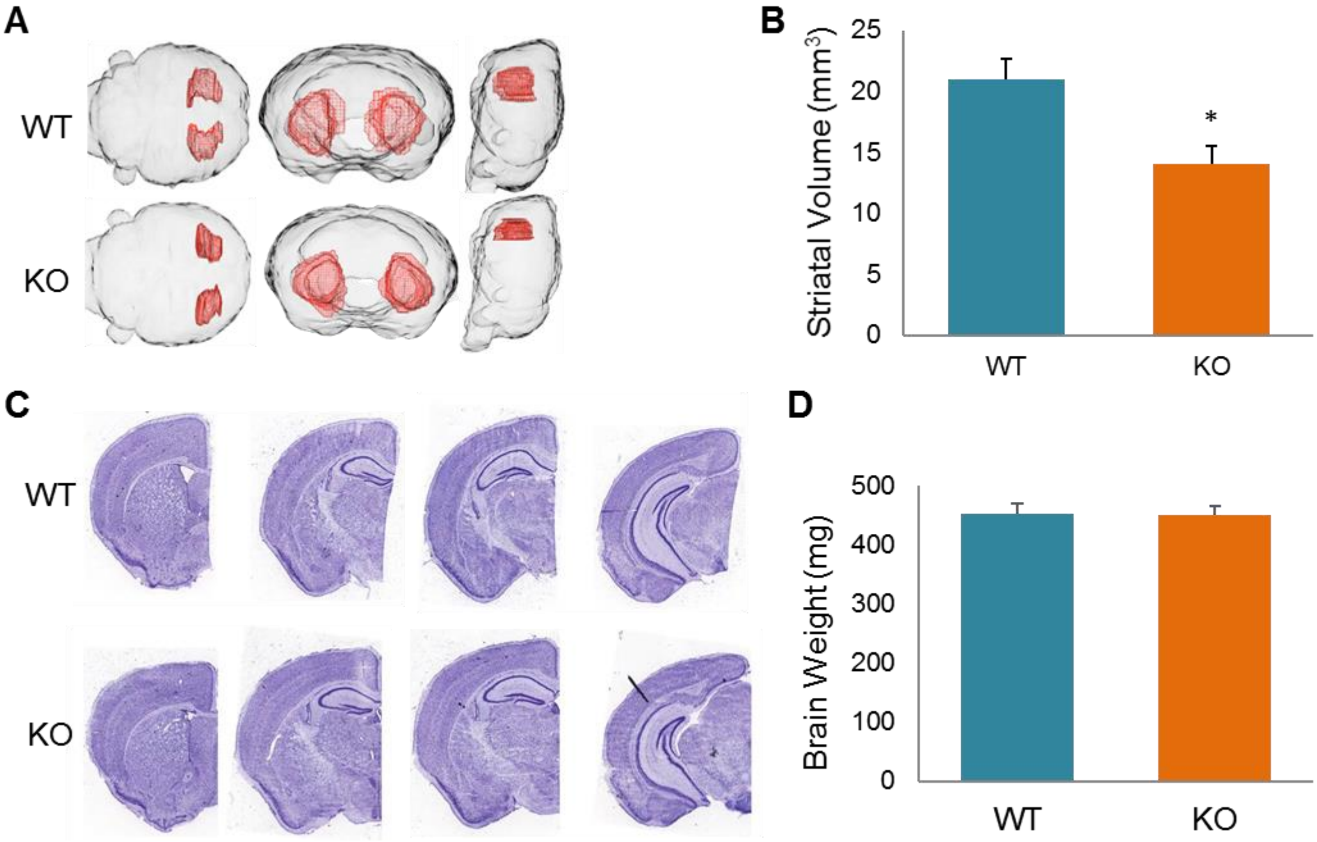

Supplementary Figure 4 Neuroarchitecture of *Phf8* KO mice. (A) Representative 3D reconstruction of MRI images taken from *Phf8* WT and KO mice (n=3); striatum is shown in red. **(B)** Quantification of striatal volume observed by MRI. *=$p<0.05$, (Student's T-test, 2-tailed) **(C)** Representative Nissl stains of sections through the brain of *Phf8* WT (top) and KO (bottom) mice. No significant differences were observed. **(D)** Weights of brains from *Phf8* WT and KO mice show no difference (Student's T-test, 2 tailed). Error bars=s.e.m.

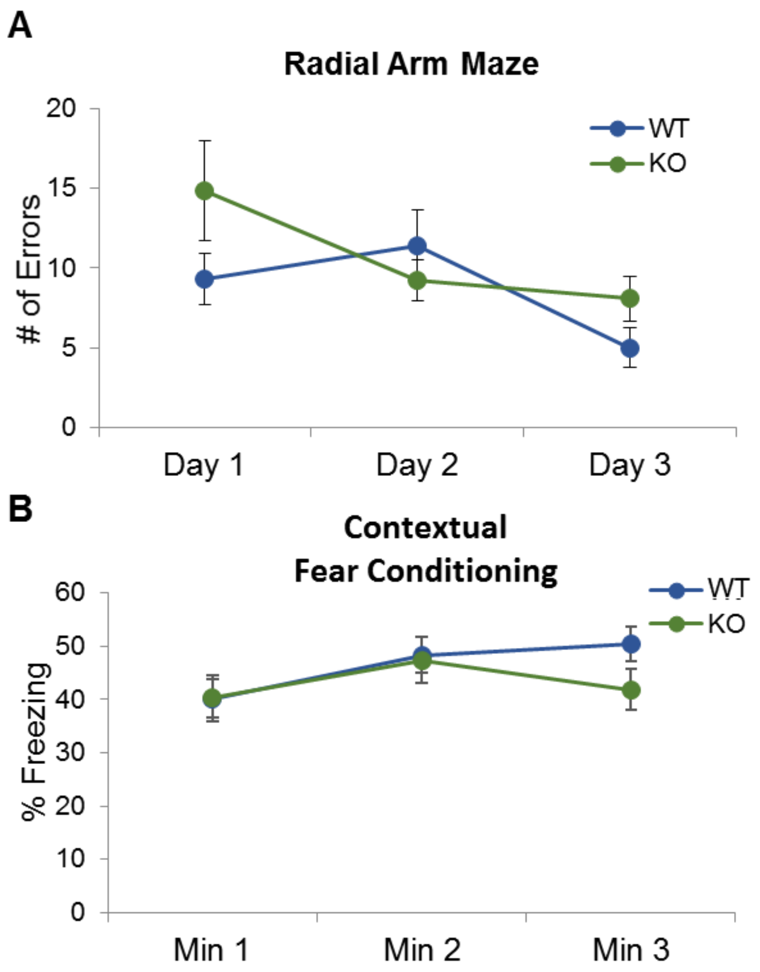

Supplementary Figure 5 *Phf8* null mice do not display signs of intellectual disability. (A) Number of errors (multiple entries into the same arm) made in the radial arm maze test. No significant difference observed (Student's T-test, 2-tailed) **(B)** Freezing behavior observed during testing phase of contextual fear conditioning. No significant difference observed (Student's T-test, 2-tailed). Error bars=s.e.m.

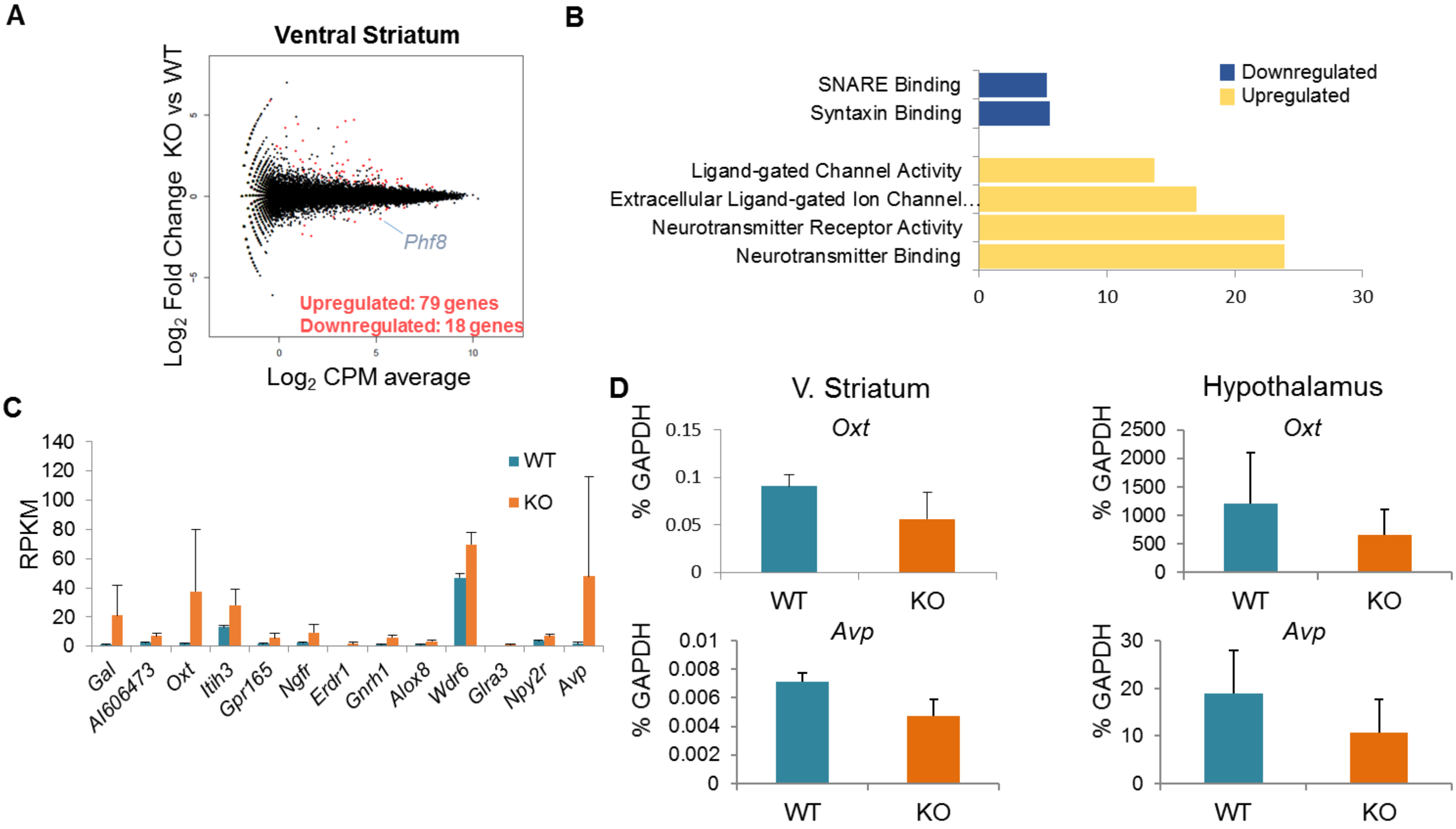

Supplementary Figure 6 Differentially expressed genes in the ventral striatum of *Phf8* null mice. (A) Mean Average plots of RNA-seq data from the ventral striatum comparing *Phf8* WT and KO mice. Differentially expressed genes ($p<0.05$, $FDR<0.05$) between WT and KO mice for each sample set are shown in red. **(B)** Gene ontology (GO) molecular function analysis of the differentially expressed upregulated and downregulated gene sets from the ventral striatum (1.4 fold cutoff, $FDR<0.05$). **(C)** RPKM values for the most significantly (ranked by p-value) upregulated genes from the ventral striatum. **(D)** RT-qPCR for two hypothalamic genes, *Oxt* and *Avp*, linked to anxiety and upregulated in the RNA-seq data from the *Phf8* KO mice. Note that we failed to detect increased expression of these genes in the ventral striata from a new set of mice and in the hypothalamus (Student's T-test, 2 tailed), where these genes are normally expressed (WT: n=3, KO: n=5), suggesting hypothalamic contamination of the original *Phf8* KO ventral striata owing to the reduced striatal size in the KO mice. Error bars=s.e.m.

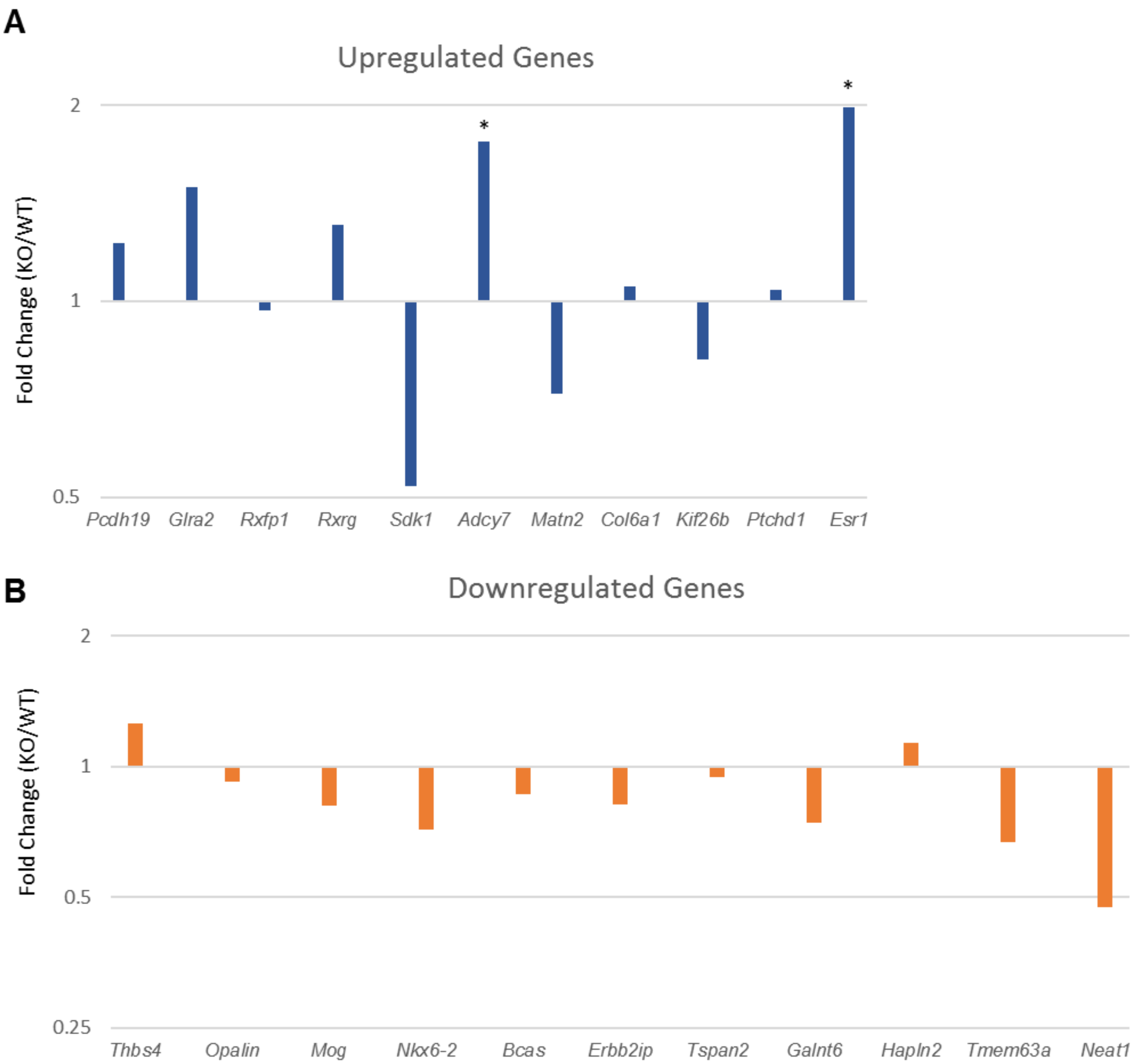

Supplementary Figure 7 Validation of RNA-seq results. (A&B) RT-qPCR analysis of most significantly (based on p-value) upregulated **(A)** and downregulated **(B)** genes, excluding *Htr1a, Htr1b* & *Htr2a*, which are shown in Fig. 5E. RNA was isolated from PFCs of mice not used for RNA-seq. n=3 for WT, n=4 for KO *=p<0.05, (Student's T-test, 2-tailed).

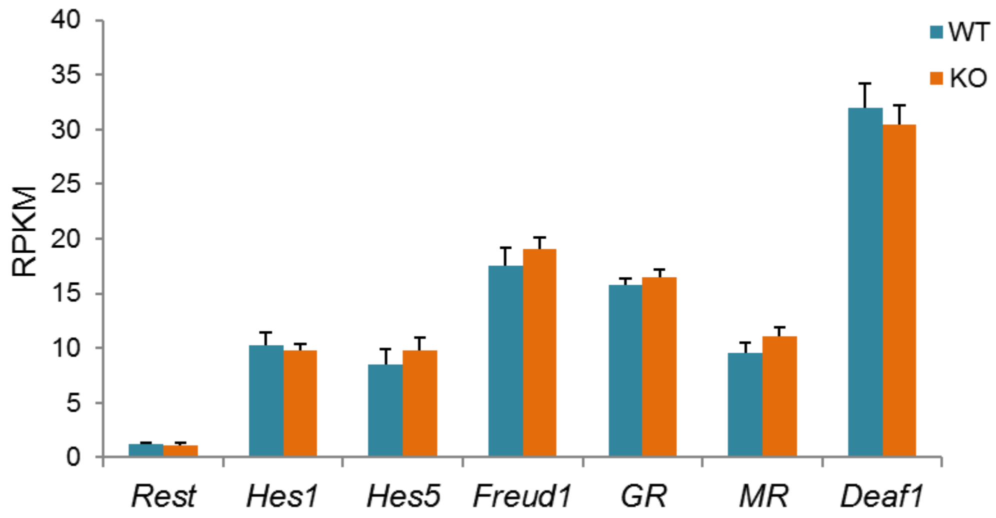

Supplementary Figure 8 Known repressors of *Htr1a* are not missexpressed in *Phf8* KO mice. RPKM expression values for known transcriptional regulators of *Htr1a* from RNA-seq of *Phf8* WT and KO prefrontal cortices. No difference in expression was observed between *Phf8* WT and KO samples (Student's T-test, 2 tailed). Error bars=s.e.m.

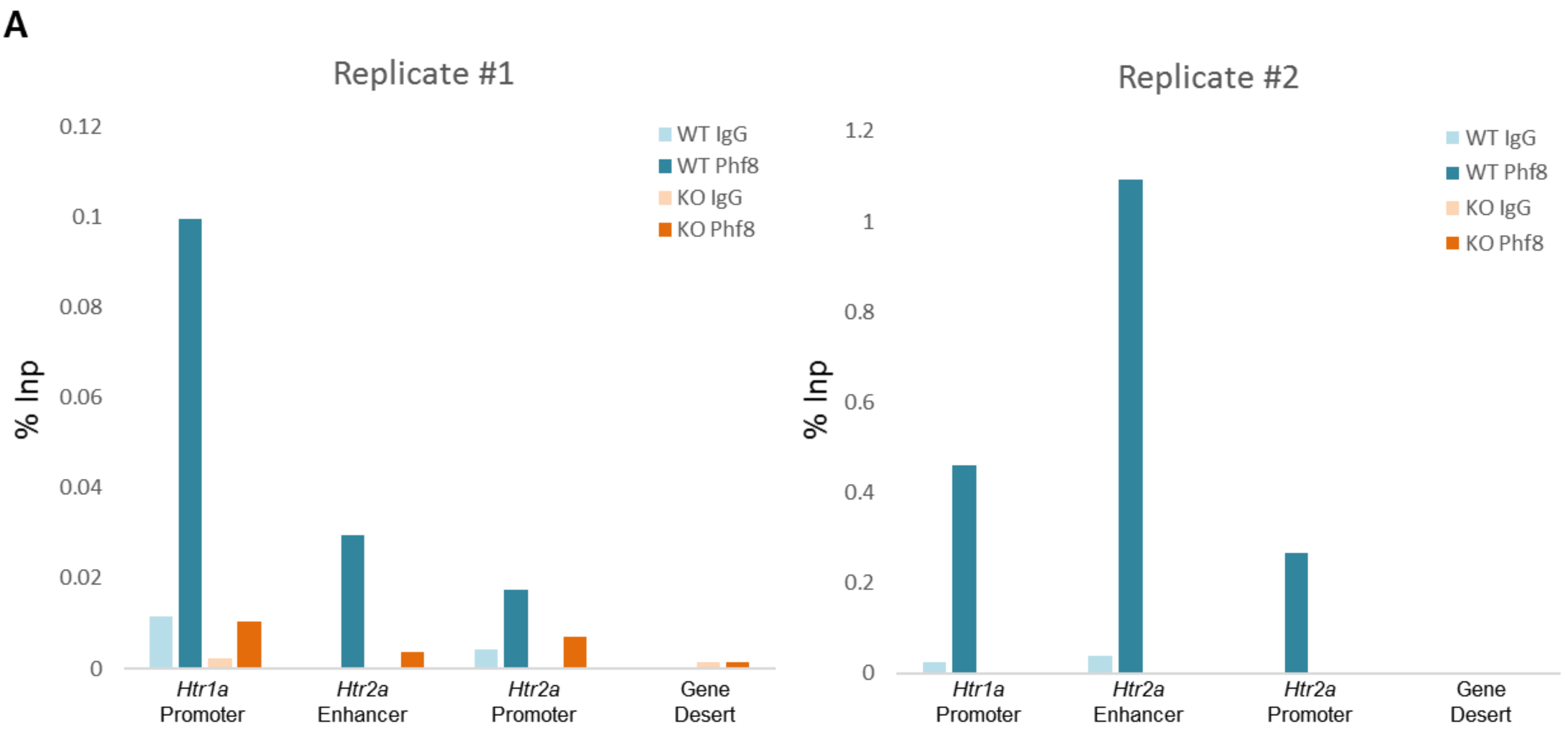

Supplementary Figure 9 Replicates of Phf8 ChIP-qPCR analysis at *Htr1a* & *Htr2a* loci. Replicate ChIP-qPCR analyses pertaining to representative experiment shown in Figure 6B. Each replicate was carried out independently on mice from separate litters and analyzed via qPCR as described in Figure 6B.

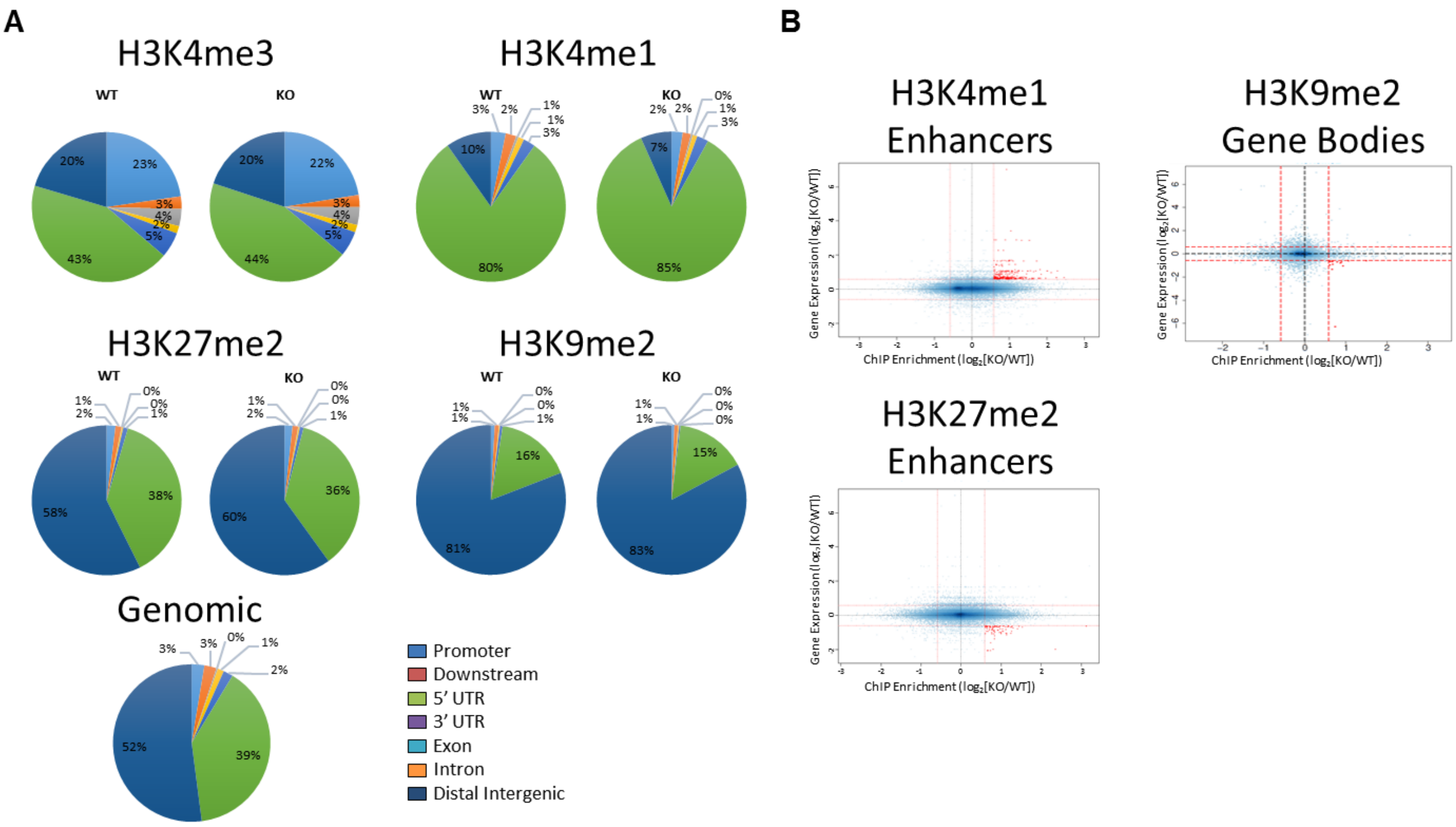

Supplementary Figure 10 ChIP-seq analysis for histone modifications in neocortical neurons. (A) Relative enrichment profiles for the histone modifications H3K4me1, H3K4me3, H3K9me2 and H3K27me2 in WT and KO neurons. promoter=TSS-3kb, downstream=TTS+3kb. **(B)** Relative gene expression levels from PFC RNA-seq versus relative histone modification enrichment either at enhancers for H3K4me1 (top left) and H3K27me2 (bottom left) or within gene bodies for H3K9me2 (top right).

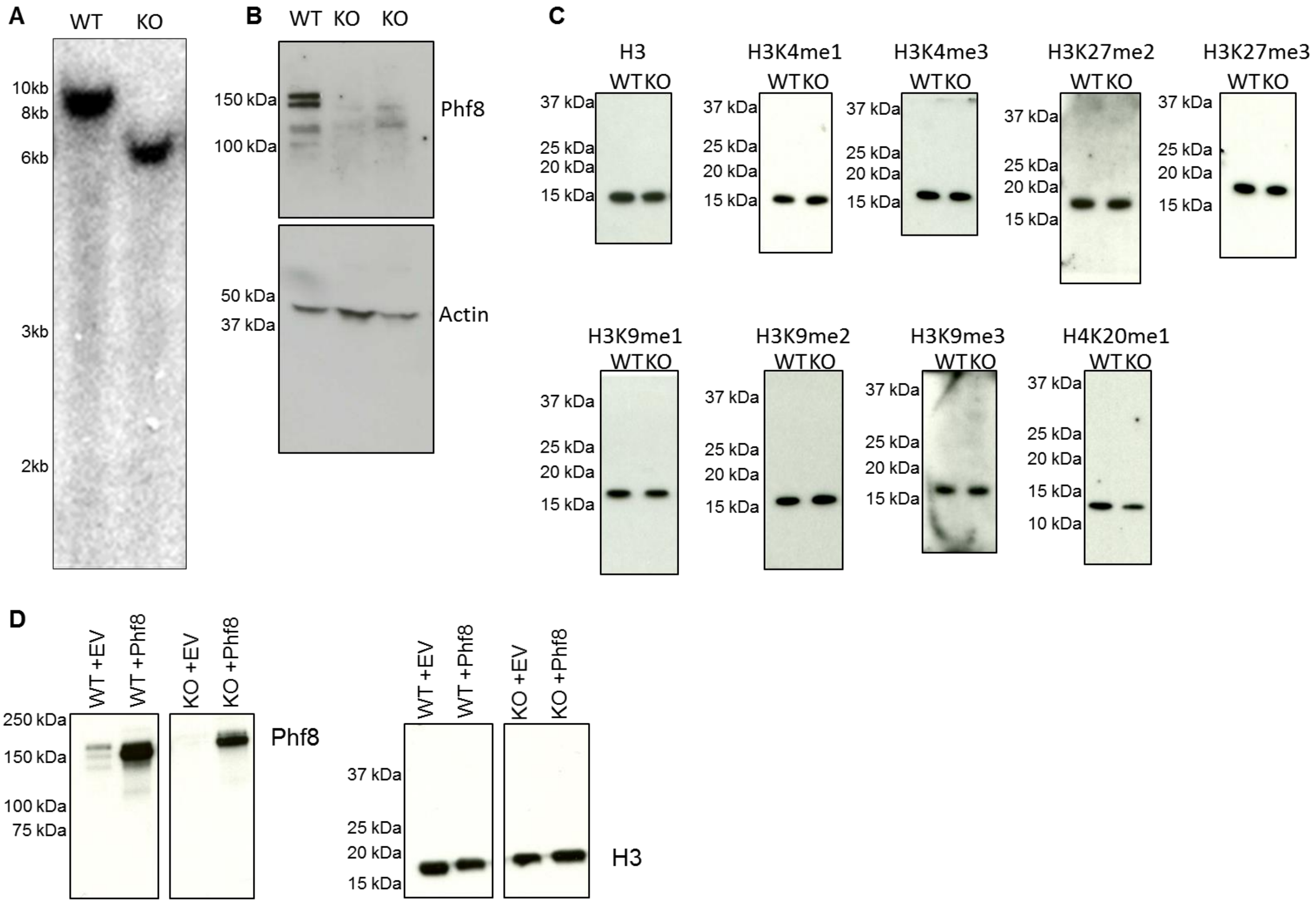

Supplementary Figure 11 Uncropped Southern and Western blots. (A) Southern blot from Figure 1B. **(B)** Western blot from Figure 1C. **(C)** Western blots from Figure 1E. **(D)** Western blots from Supplementary Figure 1C.

Phf8$^{+/-}$ x *Phf8*$^{+/y}$

Genotype	*Phf8*$^{+/+}$ and *Phf8*$^{+/y}$	*Phf8*$^{-/+}$	*Phf8*$^{-/y}$
Total	84	46	41
% Observed	49.1	26.9	34
% Expected	50	25	25

Supplementary Table 1. Phf8 KO mice are born at Mendelian ratios. Genotypes of live mice obtained from a cross between a *Phf8*$^{+/-}$ female and a *Phf8*$^{+/Y}$ male mice. Note that *Phf8* KO mice are born at the expected Mendelian ratios

Assay	Behavior Assessed	# of mice	Phenotype in KO animal	Figure
Forced Swim	Depression-like	WT n=17<br>KO n=16	↑ Latency to immobile<br>↓ time immobile	Fig. 3b&c
Tail Suspension	Depression-like	WT n=17<br>KO n=16	Not detected	Fig. 3e&ef
Social Defeat	Depression-like	WT n=7<br>KO n=9	↑ Time in interaction zone	Fig. 3h&i
Open Field Locomotion	Anxiety-like	WT n=17<br>KO n=16	↑ Activity in new environment	Fig. 2b
Center Entry	Anxiety-like	WT n=17<br>KO n=16	↑ Entries into center<br>↑ Time in center	Fig. 2c
Elevated Plus Maze	Anxiety-like	WT n=17<br>KO n=16	↑ Time in open arms	Fig. 2e&f
Light/Dark Box	Anxiety-like	WT n=17<br>KO n=16	Not Detected	Fig. 2h&i
Radial Arm Maze	Working Memory	WT n=12<br>KO n=13	Not Detected	Suppl. Fig. 5a
Contextual Fear Conditioning	Associative Learning	WT n=30<br>KO n=30	Not detected	Suppl. Fig. 5b

Supplementary Table 2. Behavioral Assessment of Phf8 KO mice.

Primer	Sequence
Avp RT-qPCR F101	GCTGCCAGGAGGAGAACTAC
Avp RT-qPCR R101	AAAAACCGTCGTGGCACTC
Htr1a ChIP En 118F	TCTAAATGGCGCTCTGAAGC
Htr1a ChIP En 118R	CATCCCTGTCTCCAGCAACT
Htr1a ChIP Prom 119F	AGGTAAGAGGCGGGGTTTAG
Htr1a ChIP Prom 119R	ACATTCCAGTCCACACCACA
Htr1a RT-qPCR F108	TGAAGACACTGGGCATCATC
Htr1a RT-qPCR R108	TTATGGCACCCAACAACTCA
Htr1b ChIP En1 120F	TGAAACTTGGAGTCGCCTTT
Htr1b ChIP En1 120R	AGCGCAAAGACTCAAAGCTC
Htr1b ChIP En2 121F	CCCATCCTCCAGTCTTCAAC
Htr1b ChIP En2 121R	AGCTTCCAGTCCGTTTTCAA
Htr1b ChIP Prom 122F	GGGAGGATTGAATGACAAGC
Htr1b ChIP Prom 122R	GGGGCTTTGAGACATCTTTG
Htr1b RT-qPCR F109	TCTCCCTGGTGATGCCTATC
Htr1b RT-qPCR R109	TGTGGAACGCTTGTTTGAAG
Htr2a ChIP En 123F	GGCCTCTTTGGCTGAGAGTA
Htr2a ChIP En 123R	CCCTGCTGGACTGTAGTGCT
Htr2a ChIP Prom 128F	CCTGGACACATCATCACTGG
Htr2a ChIP Prom 128R	GCTGAGGGGTGAAGAATGAG
Htr2a RT-qPCR F110	ATAGCCGCTTCAACTCCAGA
Htr2a RT-qPCR R110	TCATCCTGTAGCCCGAAGAC
Otx RT-qPCR F100	GCCAGGAGGAGAACTACCTG
Otx RT-qPCR R100	CTCCGAGAAGGCAGACTCAG
Phf8 Ext Probe 7F	TTATCCCCAAGTCCCCCTAT
Phf8 Ext Probe 7R	CAGGAAGGACCATCCAGAGA
Utf1 ChIP Prom 35F	TCCTGGGTCCCTAAGGAAAG
Utf1 ChIP Prom 35R	CCCTCGCCTACCTAGTTCCT
Gene Desert ChIP F1	TGGGTGCCGTATGCCACATTAT
Gene Desert ChIP R1	TTTCTGGCCATCCGCACCTTAT
Mac1 ChIP prom F1	GATCCGAAACACTGAGCCTACT
Mac1 ChIP prom R1	TGAACTGTGGTATTTCAGGCAC

Supplementary Table 3. Primer sequences used in this study
